# Retraction: Overexpression of long non-coding RNA SBF2-AS1 promotes cell progression in esophageal squamous cell carcinoma (ESCC) by repressing miR-494 to up-regulate PFN2 expression

**DOI:** 10.1242/bio.048793retraction

**Published:** 2025-01-24

**Authors:** Qiu Zhang, Xixiang Pan, Dongyang You

The journal is retracting *Biol. Open* (2020), bio048793 (doi:10.1242/bio.048793) because of concerns with reliability of the data.

After publication of the accepted manuscript in 2020, concerns were raised about the integrity of the western blots presented in this paper. The journal asked the authors to supply original blot data but did not receive a response, despite the accepted manuscript having been published very recently. Owing to the unavailability of raw data, this article fails to comply with our data retention policy. The journal has lost confidence in the reliability of results presented in this study and is therefore retracting this article. The original article is available in the supplementary information to this Retraction notice.

The authors did not respond to say whether they agree with this retraction.

## Supplementary Material

10.1242/biolopen.048793_sup1 Supplementary information

