# Supplementary material for: Retraction: Overexpression of long non-coding RNA SBF2-AS1 promotes cell progression in esophageal squamous cell carcinoma (ESCC) by repressing miR-494 to up-regulate PFN2 expression
Source: Biol Open. 2025 Jan 24;14(1):bio048793. doi: 10.1242/bio.048793retraction (PMC11968086; doi:10.1242/bio.048793retraction)
Supplement: Supplementary information [file biolopen-14-048793-s1.pdf]

RETRACTED: Overexpression of long non-coding RNA SBF2-AS1 promotes cell progression in esophageal squamous cell carcin[illegible] (ESCC) by repressing miR-494 to up-regulate PFN2 expr[illegible]sion

Qiu Zhang[#,1], Xixiang Pan[#,1], Dongyang You[*,1]

[1]Oncology Department of Thoracic Head and Neck S[illegible]al, Huangshi [illegible]entral Hospital (Affiliated Hospital of Hubei Polytechnic University), Edong Hea[illegible]are Group, Huangshi, Hubei, China

[#]Qiu Zhang and Xixiang Pan contribute[illegible]lly to this work.

[*]**Corresponding author:** Do[illegible]gyang [illegible]ou, Onc[illegible]ogy Department of thoracic head and neck surgical, Huangshi Central [illegible]spit[illegible] (Affiliated Hospital of Hubei Polytechnic University), Edong Healthcare Gr[illegible]p, No. 293, Yi[illegible]n Street, Huangshi, 435000, Hubei, China

E-mail: zuibizhi19821@126.[illegible]m Tel.: 86-0714-3189999

RETRACTED

© 2020. Published by The Company of Biologists Ltd.
This is an Open Access article distributed under the terms of the Creative Commons Attribution License (http://creativecommons.org/licenses/by/4.0), which permits unrestricted use, distribution and reproduction in any medium provided that the original work is properly attributed.

Biology Open • Accepted manuscript

ABSTRACT

Esophageal squamous cell carcinoma (ESCC) is an intractable esophageal cancer caused by smoking, alcohol consumption and nutritional deficiencies. Recently, long non-coding RNA SET-binding factor 2 antisense RNA 1 (SBF2-AS1) was validated as an oncogene in multiple cancers. However, the mechanism of SBF2-AS1 in ESCC progression is poorly understood. In the present research, we found that the expression of SBF2-AS1 and PFN2 was up-regulated, while miR-494 was down-regulated in ESCC tumors and cells using quantitative real-time polymerase chain reaction (qRT-PCR). 3-(4, 5-dimethyl-2-thiazolyl)-2, 5-diphenyl-2-H-tetrazolium bromide (MTT) assay and transwell assay demonstrated that silencing of SBF2-AS1 suppressed proliferation, migration and invasion. Moreover, western blot showed that SBF2-AS1 deletion [illegible] inhibited epithelial to mesenchymal transition (EMT) by detecting MMP9, Vimentin and E-cadherin protein expression. We confirmed that miR-494 was a target of SBF2-AS1 by luciferase reporter system, RIP and RNA pull-down assay. In addition, miR-494 inhibitor reversed the repression induced by SBF2-AS1 silencing on ESCC cell proliferation, migration, invasion and EMT. Furthermore, PFN2 was negatively regulated by miR-494. Besides, restoration of PFN2 inversed the inhibition effects on cell proliferation, migration, invasion and EMT induced by SBF2-AS1 silencing in ESCC. In conclusion, SBF2-AS1 contributed to cell proliferation, migration, invasion and EMT in ESCC by enhancing PFN2 expression via sponging miR-494, providing promising biomarkers for ESCC diagnosis and treatment.

Keywords: SBF2-AS1, miR-494, PFN2, progression, ESCC

RETRACTED

Biology Open • Accepted manuscript

RETRACTED

INTRODUCTION

Esophageal squamous cell carcinoma (ESCC) which originated from esophageal epithelial cells, accounts for over 90% esophageal cancers (Hou et al., 2018; Kang [illegible] al., 2018). The major pathogenic factors include smoking, alcohol consumption and nutritional deficiencies (Guo et al., 2018). Despite advanced interventions, such as optimal surgery followed by chemotherapy, the 5-year survival rate is approximately 15% since distant metastasis, high aggressiveness and locoregional recurrence (Kawaguchi [illegible] al., 2017; Nish[illegible]mi et al., 2019; Wu, Yang, & Xian, 2019). Therefore, illumination of the underlying pathogenesis of ESCC is urgently needed.

Long noncoding RNAs (lncRNAs) are conserved transcripts that functionally participate in cell regulation of many diseases, especially cancers (L. Chen et al., 2019; Hong, Chen, Wu, & Wang, 2018; X. Hu, Ding, Zhang, & Cu[illegible] 2019). LncRNA SET-binding factor 2 antisense RNA 1 (SBF2-AS1) mapped on human chromosome 11p15.1, was a newly identified oncogene in multiple cancers such as small-cell lung cancer, cervical and colorectal cancer (Y. Zhang, Li, Han, Zhang, & Sun, 2019). As competing endogenous RNA (ceRNA), SBF2-AS1 accelerated cell progression in acute myeloid leukemia by targeting miR-188-5p (Y. J. Tian et al., 201[illegible]). Moreover, overexpression of SBF2-AS1 enhanced cell proliferation and invasion [illegible] colorectal cancer by sponging miR-619-5p to up-regulate HDAC3 expression (G. Chen [illegible] al., 201[illegible]). Chen and his colleagues reported that SBF2-AS1 upregulation was observed in ESCC (Chen et al., 2018). LncBase Predicted v.2 predicted that microRNA-494 (miR-494) was a target gene of SBF2-AS1. MiR-494, a small chain noncoding RNA, was demonstrated to function as tumor promoter or suppressor in tumorigenesis and progression

Biology Open • Accepted manuscript

in cancers (J. Zhang et al., 2019; Cheng et al., 2018; N. Li et al., 2016). However, the functional role and the regulatory mechanism of SBF2-AS1/miR-494 axis were still unknown.

Profilin 2 (PFN2) is a member of profiling family that exclusively [illegible] the central nervous system. PFN2 was also closely related to cell [illegible] migra[illegible]on in a variety of cancers (Jeong, Choi, Seo, Lee, & Yoo, 2018). In [illegible] recognized as essential unfavorable prognosis biomarker of different [illegible], including [illegible] (Cui et al., 2016). Therefore, the function of PFN2 in ESCC requires in-depth [illegible].

We aimed to illuminate the regulatory [illegible] of SBF2-[illegible] on ESCC cell progression. The expression of SBF2-AS1, miR-494 and [illegible] was [illegible]. Moreover, loss-of-function experiment was conducted to [illegible] effects of SBF2-AS1 on cell proliferation, migration, invasion and EMT of [illegible] cells. Besides, the influence of SBF2-AS1/miR-494/PFN2 [illegible] on [illegible] ESCC cells was also investigated.

MATERIALS AND [illegible]

Tissue samples

ESCC [illegible] (n=[illegible] were recruited from Huangshi Central Hospital (Affiliated Hospital of Hubei [illegible]). All the patients were informed and signed informed consent. [illegible] tissues [illegible]=50) and normal tissues (n=15) were obtained from those patients by [illegible] subjected to the following experiments. Our protocols were approved by Ethics [illegible] of Huangshi Central Hospital (Affiliated Hospital of Hubei Polytechnic University).

RETRACTED

Biology Open • Accepted manuscript

RETRACTED

Cell culture and transfection

Eca-109, KYSE-150, TE-1 cells were purchased from Chinese Academy of Sciences (Shanghai, China), KYSE-140 cells were purchased from Bioleaf (Shanghai, China) and human esophageal epithelium immortalized cells HET-1A were purchased from American Type Culture Collection (ATCC, Manassas, VA, USA). All the cells were maintained in DMEM medium (Gibco, Carlsbad, CA, USA) supplemented with 1[illegible]BS (Gibco) and 0.05% penicillin/streptomycin.

PFN2 overexpression vector was obtained by cloning the sequences of PFN2 (423 bp, GeneBank accession No. KM517577) (GenePharma, Shanghai, China) into pcDNA3.1 (Invitrogen, Carlsbad, CA, USA), termed pcDNA3.1-PFN2 (PFN2). Before transfection, Eca-109 and KYSE-150 cells (4 × 10^5 [illegible] per well) were seeded into 12-well plate. After incubation for 24 h, 0.2 μg of PFN2 overexpression vector (PFN2) were transfected into Eca-109 and KYSE-150 cells using 0.5 μL of Lipofectamine 2000 reagent (Invitrogen). Moreover, those oligonucleotides included that small harboring RNA (shRNA) targeting SBF2-AS1 (2,708 bp, GeneBank accession No. NR_036485) (sh-SBF2-AS1) and the negative control (sh-NC) were synthesized by Genepharma (Shanghai, China), and miR-494 mimics and negative control (miR-NC) were purchased from RIBOBIO (Guangzhou, China). Eca-109 and KYSE-15[illegible] cells were transfected with 0.5 μg of aforementioned oligonucleotides using 0.6 μL of Lipofectamine 2000 (Invitrogen). After transfection for 48 h, the transfected cells were used for further analysis.

Biology Open • Accepted manuscript

RETRACTED

Quantitative real-time polymerase chain reaction (qRT-PCR)

QRT-PCR was performed following the method described in our earlier study (Y. J. Tian et al., 2019). Total RNA was obtained by incubating ESCC tissues and cells wi[illegible] TRIzol r[illegible]ent (Invitrogen). Then, a NaniDrop ND-1000 Spectrophotometer (NaniDrop, [illegible]mington, [illegible]A, USA) was used to quantify RNA, and its purity was detected [illegible]sing th[illegible] A260/2[illegible] ratio. Reversely transcription assay (5 µg RNA) was carried out [illegible]ynthesiz[illegible] cDNA by All-in-One™ First-Strand cDNA Synthesis Kit (FulenG[illegible]uangzhou, C[illegible]na). Briefly, the transcription was conducted in a 10 µL reaction mixture, including p[illegible]yadenylated RNA (100 ng), 5 × PrimeScript Buffer (2 µL), PrimeScr[illegible]t RT Enzy[illegible]e Mix I (0.5 µL), RT primer mixure (1 µL), and RNase-free water. Wherea[illegible] total rea[illegible]on mixture was incubated at 50°C for 15 min and 85°C for 5 sec[illegible] qRT-PCR was performed using SYBR green (Applied Biosystems, Foster City, CA, U[illegible]A) in 15 µL final volumes containing 1.5 µL template cDNA mixed with [illegible]5 µL [illegible]×SYB[illegible]Green PCR master mix and 3 µL of each forward and reverse prime[illegible]GA[illegible]DH and U6 were exploited as internal reference. The relative expression o[illegible]HOXA10 and [illegible]R-128/miR-142 was calculated using $2^{-\Delta\Delta Ct}$ method. The amplification paramete[illegible]ere as follows: denaturation at 95°C for 10 min, followed by 40 cycles of[illegible]enatur[illegible]on at 95°C for 30s, annealing at 60°C for 30s and extension at 72°C for 1min. The p[illegible]s for SP[illegible]2-AS1, miR-494, PFN2 GAPDH, and U6 were listed in Table S1. Th[illegible]fold c[illegible]nges of i[illegible]dividual genes were detected by $2^{-\Delta\Delta Ct}$ methods (VanGuilder, Vrana, & [illegible]ma[illegible] 2008).

Biology Open • Accepted manuscript

3-(4, 5)-dimethylthiazole-2-y1)-2, 5-biphenyl tetrazolium bromide (MTT)

Transfected Eca-109 and KYSE-150 cells (5 × 10^4 cells per well) were inoculated on 96-well plates and incubated for 24 h, 48 h and 72 h. Then, 10 μL MTT (Beyotime, [illegible]hanghai, C[illegible]ina) was added to each well at the different time points (0 h, 24 h, 48 h, 72 h) [illegible]r 4 h at 3[illegible]C, followed by incubating with 100 μL dimethyl sulfoxide (DMSO; [illegible]angon, [illegible]hanghai, China) for 2 h. Finally, the OD value (490 nm) was measured by a spec[illegible]otomet[illegible] (Xia et al., 2020).

Transwell assay

Cell migration and invasion were determi[illegible] using T[illegible]nswell chambers (24-well, Sigma-Aldrich). The upper chamber of [illegible]ell was treated with Matrigel (without Matrigel coating for migration assay). In briefly, t[illegible]cted Eca-109 and KYSE-150 cells (7 × 10^4 cells per well) were resusp[illegible]nded [illegible] serum[illegible]ee DMEM (Gibco). Then, the medium containing the cells were se[illegible]d i[illegible] the upper chamber. Meanwhile, the lower chamber was supplemented with [illegible] medium co[illegible]ning 10% FBS (Gibco) as chemoattractant. After incubation for 48 h, the [illegible]-migrated and non-invasive cells were scraped, while the migrated a[illegible] invade[illegible] cells at the lower chamber of transwell were fixed with methsnol and stained wit[illegible] 1[illegible] crystal [illegible]iolet (Sigma, St. Louis, MO, USA). The number of migrated and inv[illegible]ded ce[illegible]s was cou[illegible]ted using a microscope (Lv et al., 2016).

Western [illegible] assay

PFN2, N-cadherin, Vimentin and E-cadherin protein were isolated from transfected Eca-109

RETRACTED

Biology Open • Accepted manuscript

and KYSE-150 cells using pre-cold RIPA buffer reagent (Thermo Fisher Scientific, Waltham, MA, USA) containing protease inhibitor (Cocktail, Roche, Basel, Switzerland). Then, a Bicinchonnic Acid Protein Assay kit (Thermo Fisher Scientific) was used t[illegible]etect the p[illegible]tein concentration. 20 μg proteins were separated by 10% s[illegible]um dod[illegible]cyl sulphate-polyacrylamide gel electrophoresis (SDS-PAGE), tra[illegible]erred [illegible] polyvi[illegible]ylidene difluoride membranes (Millipore, Billerica, MA, USA) and block[illegible]y 5% n[illegible]fat milk at room temperature for 1.5 h. Then, the membranes were [illegible]ubated with pr[illegible]mary antibodies against PFN2 (1:1000 dilution, ab230673, Abcam, Cambridge, [illegible]A, USA), N-cadherin (1:1000 dilution, ab18230, Abcam), Vimenti[illegible] (1:1000 di[illegible]tion, ab18230, Abcam) and E-cadherin (1:1000 dilution, ab76055, Abcam) o[illegible]rnight at 4[illegible], followed by incubating with HRP-conjugated secondary antibody ([illegible]) for 1 h at 37°C. Finally, the protein signals were determined using an electrochemi[illegible]escent system (PerkinElmer Life Science, Waltham, MA, USA) (Wang e[illegible]al., 20[illegible]).

Subcellular localiza[illegible]n

The localization o[illegible] SBF2-A[illegible]was detected by cytoplasmic & Nuclear RNA Purification Kit (Norgen Bi[illegible]ek Corp[illegible] Belmont, MA, USA). Briefly, cells were lysed and then centrifugated. Subsequent[illegible] cytopl[illegible]mic RNA and nuclear RNA were incubated with Buffer SK and an[illegible]drous [illegible]thanol, re[illegible]pectively. Then, the nuclear RNA and cytoplasmic RNA were eluted [illegible] t[illegible] spin column. Finally, the expression of SBF2-AS1 in cytoplasmic and nucleus fractions [illegible]detected by qRT-PCR (Zhou et al., 2019).

Biology Open • Accepted manuscript

Luciferase reporter assay

LncBase Predicted v.2 predicted that miR-494 was a target gene of SBF2-AS1 and starBase 2.0 predicted that PFN2 was a target gene of miR-494. Based on these [illegible]ediction r[illegible]ults, SBF2-AS1 sequence (2,708 bp, GeneBank accession No. NR_036485) [illegible] PFN2 3’[illegible]TR sequence (423 bp, GeneBank accession No. KM517577) containin[illegible] wide-t[illegible]e or mu[illegible]nt-type miR-494 (location: chr14, 101029681-101029702. putative binding [illegible]ence: [illegible]ACAAAG or ACAAAG) targeting sites were cloned into pmi[illegible]O vector (Pro[illegible]ega, Madison, Wisconsin, USA), termed WT-SBF2-AS1 or MUT-SBF2-AS1, a[illegible] PFN2 3’UTR-WT or PFN2 3’UTR-MUT reporter plasmids. Wild ty[illegible]e (WT-SBF[illegible]AS1, PFN2 3’UTR-WT) and mutant type (MUT-SBF2-AS1, PFN2 3’UTR-[illegible]UT) lucifer[illegible]e vectors were constructed. DNA sequencing was used to detect th[illegible]ters. Then, 400 ng of the constructed plasmids, 50 ng of renilla luciferase reporter plasmi[illegible] (p[illegible]L-TK) and 50 nM of miR-NC and miR-494 were transfected into Eca-10[illegible] and K[illegible]SE-150 [illegible]ells using Lipofectamine 3000 (Invitrogen). After 48 h incubation, a Mi[illegible]pla[illegible] reader (Biotek Instruments) and dual luciferase reporter assays kit (Promega) [illegible]ere used to de[illegible]ine the relative luciferase activity. Renilla luciferase activity was regarded as the [illegible]rnal control for the normalization of firefly luciferase activity (X. Rong et [illegible]., 2019[illegible]

RN[illegible] imm[illegible]noprecip[illegible]ation (RIP) assay

[illegible] as[illegible]y was carried out using RIP™ RNA-Binding Protein Immunoprecipitation Kit (Millipore[illegible] [illegible]Wu et al., 2019). Firstly, 2×10^5 Eca-109 and KYSE-150 cells were lysed by RIP buffer. Then, the cell lysis was incubated with magnetic beads coated with Anti-Ago2 or

RETRACTED

Biology Open • Accepted manuscript

Anti-IgG antibody (Millipore). The enrichment of SBF2-AS1 was analyzed by qRT-PCR.

RNA pull-down assay

A pull-down assay was performed as previously described (Gao et al., 2019). Briefly, 2×10^5 Eca-109 and KYSE-150 cells were collected, lysed, and sonicated. Biotinylated wild type miR-494 (Bio-miR-494-WT), mutant type miR-494 (Bio-miR-494-MUT) and negative control (Bio-NC) synthesized by RiboBio (Guangzhou, China) were transfected into Eca-109 and KYSE-150 cells. Then, Eca-109 and KYSE-150 cells were incubated with Dynabeads M-280 Streptavidin (Invitrogen) for 10 min. After washing with wash buffer, the RNA complexes bound to the beads were eluted and isolated using the RNeasy Mini Kit (QIAGEN) for RT-PCR.

Statistical analysis

Data were presented as mean ± standard deviation (SD). Statistical analysis was performed by GraphPad Prism [illegible]. The survival curve was plotted by Kaplan-Meier analysis. Paired Student's *t*-test or one-way analysis of variance (ANOVA) was exploited to compare two or more samples. The correlation between miR-494 and SBF2-AS1 or PFN2 was analyzed by Pearson's correlation coefficient. *P* value less than 0.05 ($P<0.05$) was considered statistically significant.

RETRACTED

Biology Open • Accepted manuscript

RETRACTED

RESULTS

SBF2-AS1 was closely associated with poor prognosis of ESCC

Initially, the role of SBF2-AS was evaluated by measuring the expression [illegible] SBF2-AS[illegible]ing GEPIA2 (http://gepia2.cancer-pku.cn/#index) and qRT-PCR. As displaye[illegible] Fig. 1[illegible]B, SBF2-AS expression was up-regulated in ESCC tumors comp[illegible]ed wi[illegible] normal [illegible]issues. Meanwhile, SBF2-AS level was considerably higher in ESC[illegible] ce[illegible]s (TE-1[illegible]KYSE-140, KYSE-150, Eca-109) than that of human esophageal epi[illegible]um immortali[illegible]d cells HET-1A (Fig. 1D). Moreover, the survival rate was significantly reduced in [illegible]CC patients with high SBF2-AS level. By contrast, low SBF2-AS leve[illegible]esulted in r[illegible]tively high survival rate (Fig. 1C). Hence, overexpression of SBF2-AS contrib[illegible]d to poor p[illegible]gnosis of ESCC.

Depletion of SBF2-AS1 repressed cell [illegible]ol[illegible]eration, migration, invasion and EMT in ESCC

Functionally depletion of [illegible]F2-[illegible]1 was conducted to further explore the influence of SBF2-AS1 on prolif[illegible]tion, migration[illegible]vasion and EMT of ESCC cells. It was apparent that SBF2-AS1 expression was [illegible]tinctly decreased in Eca-109 and KYSE-150 cells stably transfected [illegible]th sh-[illegible]BF2-AS1 compared with sh-NC group, indicating that the transfection efficiency w[illegible]tremely [illegible]igh (Fig. 2A). Cell proliferation was repressed by SBF2-AS1 sil[illegible]cing [illegible]ig. 2B-C[illegible] Meanwhile, SBF2-AS1 silencing evidently attenuated cell migration a[illegible] inv[illegible]sion of ESCC cells (Fig. 2D-E). In addition, the expression of EMT associated protein M[illegible]9, Vimentin and E-cadherin was analyzed by western blot. As illustrated in Fig. 2F, the expression of MMP9, Vimentin was reduced whereas E-cadherin expression was

Biology Open • Accepted manuscript

boosted, demonstrating that SBF2-AS1 knockdown restricted EMT process. Collectively, depletion of SBF2-AS1 inhibited cell proliferation, migration, invasion and EMT in ESCC.

SBF2-AS1 acted as sponge of miR-494

The interaction between SBF2-AS1 and miR-494 was further de[illegible]rmined [illegible]y qRT-[illegible]CR and luciferase reporter system. Obviously, SBF2-AS1 was highl[illegible] expr[illegible] in the [illegible]ytoplasm of Eca-109 and KYSE-150 cells in comparison with nucle[illegible]larifying that S[illegible]F2-AS1 mainly exerted its function in the cytoplasm of cells (Fig. 3A-B). Bi[illegible]formatics analysis by LncBase Predicted v.2 showed the existence of [illegible]tative bindi[illegible] sites between SBF2-AS1 and miR-494 (Fig. 3C). In addition, down-regulati[illegible] of miR-49[illegible] expression was observed in ESCC tumors and cells compared with [illegible]tched normal ones (Fig. 3D-E). There was also a negative linear relation between expressi[illegible] SBF2-AS1 and miR-494 (r=-0.67, P<0.001) (Fig. 3F). What's more, mi[illegible]494 e[illegible]pression [illegible]n ESCC cells was enhanced by miR-494 transfection (Fig. 3G). Re[illegible]tion [illegible]f luciferase activity in Eca-109 and KYSE-150 cells co-transfected with [illegible]T-SBF2-AS[illegible]nd miR-494 validated the interaction between SBF2-AS1 and m[illegible]R-494 (Fi[illegible]H-I). As expected, RIP assay showed that the enrichment of SBF2-AS1 [illegible]as up[illegible]gulated in ESCC cells incubated with Anti-Ago2 compared with Anti-IgG ([illegible]). By c[illegible]trast, the enrichment of SBF2-AS1 was distinctly enhanced by Bi[illegible]miR-[illegible]4-WT co[illegible]pared with Bio-miR-494-MUT (Fig. 3K). Meanwhile, SBF2-AS1 [illegible]facilitated the expression of miR-494 in ESCC cells, suggesting SBF2-AS1 could regulate t[illegible]xpression of miR-494 (Fig. 3L). Taken together, SBF2-AS1 was a sponge of miR-494.

RETRACTED

Biology Open • Accepted manuscript

MiR-494 inhibitor restrained SBF2-AS1 silencing-induced suppression on cell proliferation, migration, invasion and EMT in ESCC

The regulatory effects of SBF2-AS1/miR-494 axis on ESCC progressi[illegible] were ass[illegible]ed. Apparently, miR-494 expression was enhanced by SBF2-AS1 knockdown [illegible]le reduce[illegible] by miR-494 inhibitor (Fig. 4A). As exhibited in Fig. 4B-C, miR-494 [illegible]nibitor [illegible]bviously [illegible]escued SBF2-AS1 silencing-induced inhibition of cell growth. C[illegible]nsis[illegible], miR-[illegible]4 inhibitor counteracted SBF2-AS1 silencing-mediated repression o[illegible]CC cell migra[illegible]on and invasion (Fig. 4D-E). Besides, the expression of protein MMP9 and V[illegible]ntin was blocked by SBF2-AS1 silencing. However, the blockag[illegible] was invers[illegible] by miR-494 inhibitor. By comparison, the expression of protein E-cadher[illegible] exhibited t[illegible]e opposite trend (Fig. 4F-G). These findings implicated that SBF2-A[illegible]ld regulate ESCC cell progression by targeting miR-494.

PFN2 was a target of miR-[illegible]

As predicted by star[illegible]e2.0, miR-49[illegible]mprised the binding sites of 3'UTR PFN2 (Fig. 5A). To disclose the fu[illegible]ctional ro[illegible]f PFN2, the expression of PFN2 mRNA and protein in ESCC tumors and [illegible]ells wa[illegible]detected. As illustrated in Fig. 5B-D, PFN2 mRNA and protein levels were up-reg[illegible] in ES[illegible] tumors compared with normal tissues. Up-regulation of PFN2 m[illegible]A an[illegible] protein e[illegible]ression was also discovered in ESCC cells in comparison with normal [illegible] (F[illegible]. 5E-F). Meanwhile, we noticed that miR-494 was negatively correlated with PFN2 (r=-0.605, [illegible]<0.001) (Fig. 5G). Importantly, luciferase activity was reduced in ESCC cells co-transfected with PFN2 3'UTR-WT and miR-494. However, there was no evident change

Biology Open • Accepted manuscript

of luciferase activity in PFN2 3'UTR-MUT transfection group (Fig. 5H-I). In addition, miR-494 transfection repressed PFN2 protein expression in ESCC cells (Fig. 5J). Also, miR-494 inhibitor rescued SBF2-AS1 silencing-induced repression o[illegible] PFN2 p[illegible]tein expression (Fig. 5K-L). All the data revealed that PFN2 was negatively regu[illegible]d by miR[illegible]94 in ESCC.

Restoration of PFN2 rescued inhibition on cell proli[illegible]tion, migratio[illegible], invasion and EMT induced by SBF2-AS1 silencing in ESCC

Subsequently, the rescue experiments were con[illegible]cted by tra[illegible]fecting sh-NC, sh-SBF2-AS1, sh-SBF2-AS1+pcDNA and sh-SBF2-AS1+PF[illegible]2 in Eca[illegible]09 and KYSE-150 cells. Apparently, PFN2 protein expression [illegible]omoted by PFN2 transfection (Fig. 6A). MTT results indicated that PFN2 counteracted [illegible]e [illegible]uppression on cell proliferation induced by SBF2-AS1 silencing (Fig. [illegible]B-C). [illegible]imultan[illegible]usly, cell migration and invasion were decreased by SBF2-AS1 [illegible]nci[illegible]g. However, PFN2 reversed the effects (Fig. 6D-E). Moreover, the prod[illegible]ion of prote[illegible] MMP9 and Vimentin was blocked by SBF2-AS1 silencing and boosted by re[illegible]ration of PFN2. By contrast, the expression of E-cadherin displayed th[illegible] opposi[illegible] trend (Fig. 6F-G). Altogether, SBF2-AS1 accelerated cell proliferation, migration, in[illegible]s[illegible]n and E[illegible]T in ESCC cells by enhancing PFN2.

[illegible]stra[illegible]on of the association between SBF2-AS1/miR-494/PFN2 axis and ESCC cell progressi[illegible]

We illustrated the interrelation between SBF2-AS1/miR-494/PFN2 axis and ESCC cell

RETRACTED

Biology Open • Accepted manuscript

proliferation, migration, invasion and EMT. SBF2-AS1 could repress miR-494 to enhance PFN2 expression and further facilitating EMT by boosting MMP9, Vimentin protein expression and blocking E-cadherin protein expression. Hence, SBF2-[illegible]1 was a[illegible] to accelerate ESCC cell proliferation, migration and invasion by promoti[illegible]EMT thr[illegible]gh regulating miR-494/PFN2 axis (Figure 7).

DISCUSSION

Growing evidences demonstrated that SBF2-AS1 was critical t[illegible]apeutic biomarker in cancers, such as lung adenocarcinoma and hepa[illegible]cellular car[illegible]oma (R. Chen et al., 2019; Y. T. Zhang et al., 2018). Differentially expression [illegible] SBF2-AS[illegible] was implicated in the growth of cancer cells. For instance, increased [illegible]sion of SBF2-AS1 expedited cell development by repressing miR-140-5p and facilitating [illegible]BR1 expression in hepatocellular carcinoma (Y. Li et al., 2018). Likewise, [illegible]BF2-[illegible]1 was u[illegible]regulated in non-small cell lung cancer, and overexpression of SBF2-AS[illegible]iti[illegible]ed cell migration, invasion and impaired apoptosis (Lv et al., 2016). Specifical[illegible] up-regulation [illegible] SBF2-AS1 was reported to facilitate cell survival in ESCC cells (Chen et al., 201[illegible] In addition, exosomal transfer of SBF2-AS1 was reported to improve ch[illegible]no-resi[illegible]ance of temozolomide against glioblastoma (Z. Zhang et al., 2019). Whether SB[illegible]1 funct[illegible]ned as oncogene to improve malignancy in ESCC requires further ex[illegible]oratio[illegible]

Ba[illegible]d on the prediction by LncBase Predicted v.2, we discovered that SBF2-AS1 could specifica[illegible]bind to miR-494. Hence, we assumed that SBF2-AS1 regulated cell behavior by targeting miR-494. Previous studies indicated that dysregulation of miR-494 was implicated

RETRACTED

Biology Open • Accepted manuscript

in different cancers, including melanoma, bladder and gastric cancer (J. Li et al., 2019; Peng et al., 2018; Z. Tian et al., 2019). For instance, increased miR-494 expression enhanced cell progression and sorafenib resistance by regulating PTEN in hepatocellular carcinoma ([illegible]u et al., 2015). Similarly, elevation of miR-494 facilitated non-small cell [illegible] cancer cell proliferation, migration and repressed apoptosis by regulation of CASP2 (Q. Zhang et al., 2019). Conversely, miR-494 attenuated breast cancer cell progress[illegible]y binding to CXCR4 via Wnt/β-catenin signaling (Song et al., 2015). Also, m[illegible]494 suppressed oral cancer cell growth by blocking HOXA10 expression (Liborio-Kimura, [illegible]g, & Chan, 2015). Furthermore, miR-494 enhanced gastric cancer cell sensitivity during TRAIL treatment via repressing surviving production (Xu et al., 2[illegible]8). Therefore, SBF2-AS1 regulated cell development by targeting miR-494 in [illegible].

We firstly investigated the role of S[illegible]F2-AS1 by online tool GEPIA2 and qRT-PCR. Up-regulation of SBF2-AS1 expressi[illegible] was observed in ESCC tumors and cells. As expected, knockdown of SBF2-AS1 [illegible] ESCC cell proliferation, migration, invasion and EMT, implicating the onc[illegible]enic role of [illegible]F2-AS1. Luciferase reporter system confirmed the interaction between miR-494 [illegible]d SBF2-AS1 or PFN2. At the same time, we discovered that the expressi[illegible]n of m[illegible]-494 was down-regulated, whereas PFN2 was up-regulated in ESCC tumors and [illegible] compare[illegible] with normal ones. Next, the molecular mechanism of ESCC cell pr[illegible]gressio[illegible] was fur[illegible]er investigated by rescue experiment. MiR-494 inhibitor alleviated [illegible]F2-A[illegible]1 silencing-induced suppression on ESCC cell proliferation, migration, invasion and EMT. [illegible]onsistently, restoration of PFN2 inversed the inhibition on cell proliferation, migration, invasion and EMT in ESCC induced by SBF2-AS1 silencing. In short, SBF2-AS1

RETRACTED

Biology Open • Accepted manuscript

accelerated cell proliferation, migration and invasion in ESCC by facilitating EMT through miR-494/PFN2 axis.

CONCLUSION

In conclusion, we clarified the specific molecular mechanism of SB[illegible]-AS1 i[illegible] ESCC tumorigenesis and progression. We discovered that SBF2-[illegible]S1 p[illegible]vely ac[illegible]lerated cell proliferation, migration, invasion and EMT in ESC[illegible]lls by sponging miR-494 and up-regulating PFN2 expression. Our study was capable of rep[illegible]enting novel targeted methods for ESCC therapy.

Funding

None.

Declaration of Interests

The authors have no [illegible]erests to disc[illegible]

Acknowled[illegible]ements

None.

Au[illegible]hor c[illegible]tributions

C[illegible]cep[illegible]alization: Qiu Zhang and Xixiang Pan; Investigation: Qiu Zhang and Dongyang You; Writing - [illegible]ginal draft: Qiu Zhang and Xixiang Pan; Writing - review & editing: Qiu Zhang and Xixiang Pan; Supervision: Dongyang You.

RETRACTED

Biology Open • Accepted manuscript

References

Chen, G., Gu, Y., Han, P., Li, Z., Zhao, J. L. and Gao, M. Z. (2019). Long noncoding RNA SBF2-AS1 promotes colorectal cancer proliferation and inva[illegible]on by inh[illegible]ing miR-619-5p activity and facilitating HDAC3 expression. *J. Ce[illegible] Physiol.* [illegible]**34**, 18688-18696. doi:10.1002/jcp.28509

Chen, L., Yang, H., Yi, Z., Jiang, L., Li, Y., Han, Q., Y[illegible]ng, [illegible]hang, [illegible], Yang, Z., Kuang, Y., et al. (2019). LncRNA GAS5 regul[illegible]redox balance [illegible]nd dysregulates the cell cycle and apoptosis in malignant melanoma cells. *J. [illegible]ncer Res. Clin. Oncol.* **145**, 637-652. doi:10.1007/s00432-018-2[illegible]20-4

Chen, R., Xia, W., Wang, S., Xu, Y., Ma, Z.[illegible]u, W., Zh[illegible]ng, E., Wang, J., Fang, T., Zhang, Q. a., et al. (2019)[illegible] noncoding RNA SBF2-AS1 is critical for tumorigenesis of early-stage lung [illegible]d[illegible]nocarcinoma. *Mol Ther Nucleic Acids* **16**, 543-553. doi:10.1016/[illegible]mtn.2[illegible]19.04.0[illegible]

Chen, R., Xia, W., Wang, [illegible]Qi[illegible]M., Yin, R., Wang, S., Xi, X., Wang, J., Xu, Y., Dong, G., et al. [illegible]018). Upreg[illegible]d long non-coding RNA SBF2-AS1 promotes proliferation in esop[illegible]eal squamous cell carcinoma. *Oncol Lett* **15**, 5071-5080. doi:1[illegible].3892/[illegible].2018.7968

Cheng, L., [illegible], B., Zh[illegible]o, Y. and Jiang, J. (2018). miR-494 inhibits cervical cancer cell pr[illegible]feration [illegible]ough upregulation of SOCS6 expression. *Oncol Lett* **15**, 3075-3080. [illegible]i:10.3892/ol.2017.7651

RETRACTED

Cui, X.B., Zhang, S.M., Xu, Y.X., Dang, H.W., Liu, C.X., Wang, L.H., Yang, L., Hu, J.M., Liang, W.H., Jiang, J.F., et al. (2016). PFN2, a novel marker of unfavorable prognosis, is a potential therapeutic target involved in esophage[illegible] squamou[illegible] cell carcinoma. *J Transl Med* **14**, 137-137. doi:10.1186/s12967-016-0884[illegible]

Gao, Z. Q., Wang, J. F., Chen, D. H., Ma, X. S., Yang, W., [illegible]e, T., [illegible]d Dang, X. W. (2018). Long non-coding RNA GAS5 antagonizes th[illegible] che[illegible]istance [illegible] pancreatic cancer cells through down-regulation of miR-1[illegible]p. *Biomed. P[illegible]armacother.* **97**, 809-817. doi: 10.1016/j.biopha.2017.10.157

Guo, W., Liu, S., Dong, Z., Guo, Y., Ding, C[illegible] Shen, S., [illegible]ang, J. and Shan, B. (2018). Aberrant methylation-mediated silencing [illegible] lncRNA C[illegible]C-276P9.1 is associated with malignant progression of esoph[illegible]quamous cell carcinoma. *Clin. Exp. Metastasis* **35**, 53-68. doi:10.1007/s10585-018-[illegible]-2

Hong, L., Chen, W., Wu, D[illegible] and [illegible]ang, Y. [illegible]018). Upregulation of SNHG3 expression associated with poo[illegible]is and enhances malignant progression of ovarian cancer. *Cancer Biom[illegible]* **22**, 367-374. [illegible]:10.3233/CBM-170710

Hou, G., Zhao, Q., Zhang, [illegible] Fan, T., Liu, M., Shi, X., Ren, Y., Wang, Y., Zhou, J. and Lu, [illegible]. (2018[illegible] Down-regulation of Rictor enhances cell sensitivity to PI3K inhibitor LY2[illegible] by bl[illegible]king mTORC2-medicated phosphorylation of Akt/PRAS40 in es[illegible]hageal s[illegible]amous cell carcinoma. *Biomed. Pharmacother.* **106**, 1348-1356. [illegible]i:10.1016/j.biopha.2018.07.075

Biology Open • Accepted manuscript

Hu, X., Ding, D., Zhang, J. and Cui, J. (2019). Knockdown of lncRNA HOTAIR sensitizes breast cancer cells to ionizing radiation through activating miR-218. *Biosci. Rep.* **39**, doi:10.1042/BSR20181038

Jeong, D. H., Choi, Y. N., Seo, T. W., Lee, J. S. and Yoo, [illegible] J. (20[illegible]8). Ubiquitin-proteasome dependent regulation of Profilin2 (P[illegible]2) by [illegible]cellular [illegible]nibitor of apoptotic protein 1 (cIAP1). *Biochem. Biophys. Res. [illegible]mun.* **5[illegible]**, 423-428. doi:10.1016/j.bbrc.2018.10.115

Kang, M., Ren, M., Li, Y., Fu, Y., Deng, M. and Li, C. (2018). E[illegible]ome-mediated transfer of lncRNA PART1 induces gefitinib resistance i[illegible] esophageal squamous cell carcinoma via functioning as a competing [illegible]ndogenous [illegible]NA. *J. Exp. Clin. Cancer Res.* **37**, 171. doi:10.1186/s13046-01[illegible]

Kawaguchi, T., Komatsu, S., Ichikawa, [illegible] Hirajima, S., Nishimura, Y., Konishi, H., Shiozaki, A., Fujiwa[illegible], H., [illegible]kamoto, [illegible]., Tsuda, H., et al. (2017). Overexpression of TRIM44 is relate[illegible] i[illegible]vasive potential and malignant outcomes in esophageal squamous [illegible]ll carcino[illegible] *Tumour Biol.* **39**, 1010428317700409. doi:10.117[illegible]/101042[illegible]7700409

Li, J., Che[illegible] J., Wa[illegible]g, S., Li, P., Zheng, C., Zhou, X., Tao, Y., Chen, X., Sun, L., Wang, A., [illegible] (201[illegible] Blockage of transferred exosome-shuttled miR-494 inhibits me[illegible]noma gro[illegible]th and metastasis. *J. Cell. Physiol.* doi:10.1002/jcp.28234

[illegible] N., [illegible]hao, X., Wang, L., Zhang, S., Cui, M. and He, J. (2016). miR-494 suppresses tu[illegible] growth of epithelial ovarian carcinoma by targeting IGF1R. *Tumour Biol.* **37**, 7767-7776. doi:10.1007/s13277-015-4603-8

RETRACTED

Biology Open • Accepted manuscript

Li, Y., Liu, G., Li, X., Dong, H., Xiao, W. and Lu, S. (2018). Long non-coding RNA SBF2-AS1 promotes hepatocellular carcinoma progression through regulation of miR-140-5p-TGFBR1 pathway. *Biochem. Biophys. Res. Commun*[illegible]**03**, 2826[illegible]32. doi:10.1016/j.bbrc.2018.08.047

Liborio-Kimura, T. N., Jung, H. M. and Chan, E. K. (2015). m[illegible]-494 r[illegible]presses H[illegible]XA10 expression and inhibits cell proliferation in oral ca[illegible]er. [illegible] *Oncol.* [illegible]1, 151-157. doi:10.1016/j.oraloncology.2014.11.019

Liu, K., Liu, S., Zhang, W., Jia, B., Tan, L., Jin, Z. and Liu, Y. ([illegible]15). miR-494 promotes cell proliferation, migration and invas[illegible]n, and inc[illegible]ased sorafenib resistance in hepatocellular carcinoma by targetin[illegible] PTEN. *O[illegible]col. Rep.* **34**, 1003-1010. doi:10.3892/or.2015.4030

Lv, J., Qiu, M., Xia, W., Liu, C., Xu, Y., [illegible]ng, J., Leng, X., Huang, S., Zhu, R., Zhao, M., et al. (2016). Hi[illegible] expr[illegible]sion of [illegible]ng non-coding RNA SBF2-AS1 promotes proliferation in non[illegible]all [illegible]ell lung cancer. *J. Exp. Clin. Cancer Res.* **35**, 75-75. doi:10.1186/s[illegible]046-016-0352[illegible]

Nishiumi, S., Fujigaki, S., [illegible]ayashi, T., Kojima, T., Ito, Y., Daiko, H., Kato, K., Shoji, H., [illegible]odama, Y., Honda, K., et al. (2019). Metabolomics-based discovery of serum biom[illegible]s to p[illegible]dict the side-effects of neoadjuvant chemoradiotherapy for es[illegible]hageal [illegible]quamous cell carcinoma. *Anticancer Res.* **39**, 519-526. [illegible]i:10.21873/anticanres.13143

RETRACTED

Biology Open • Accepted manuscript

Peng, Q.P., Du, D.B., Ming, Q., Hu, F., Wu, Z.B. and Qiu, S. (2018). MicroRNA 494 increases chemosensitivity to doxorubicin in gastric cancer cells by targeting phosphodiesterases 4D. *Cell. Mol. Biol. (Noisy-le-grand)* **64**, 62-66. [illegible]MID: 306[illegible]438

Rong, X., Gao, W., Yang, X., and Guo, J. (2019). Downregulation of [illegible] circ_000[illegible]34 restricts the proliferation and invasion of cervical [illegible]cer t[illegible]ough re[illegible]gulating miR-498/BMI-1 signaling. *Life Sci.* **235**, 116785. doi:[illegible]0.101[illegible]s.2019.[illegible]6785

Song, L., Liu, D., Wang, B., He, J., Zhang, S., Dai, [illegible] Ma, X. and [illegible]ang, X. (2015). miR-494 suppresses the progression of breast cancer in v[illegible] by targeting CXCR4 through the Wnt/β-catenin signalin[illegible] pathway. *[illegible]Oncol. Rep.* **34**, 525-531. doi:10.3892/or.2015.3965

Tian, Y. J., Wang, Y. H., Xiao, A. J., [illegible] Guo, J., Wang, T. J. and Zhao, D. J. (2019). Long noncoding RNA SBF2-AS1 [illegible] a ceRNA to modulate cell proliferation via binding with miR-188[illegible]p in a[illegible]te myel[illegible]d leukemia. *Artif Cells Nanomed Biotechnol* **47**, 1730-1737. doi:1[illegible]080[illegible]1691401.2019.1608221

Tian, Z., Luo, Y., Z[illegible] J., Hua, X., [illegible] J., Huang, C., Jin, H., Huang, H. and Huang, C. (2019). Transcription[illegible] elevation of miR-494 by new ChlA-F compound via a HuR[illegible]unB a[illegible]s inhibits human bladder cancer cell invasion. *Biochim Biophys Acta Gene[illegible]l Mech* **[illegible]862**, 822-833. doi:10.1016/j.bbagrm.2019.05.007

Va[illegible]Guild[illegible], H. D., [illegible]rana, K. E., and Freeman, W. M. (2008). Twenty-five years of [illegible]antitative PCR for gene expression analysis. *Biotechniques.* **44**, 619-626. do[illegible].2144/000112776

RETRACTED

Biology Open • Accepted manuscript

RETRACTED

Wang, H., Wu, W., Wang, H. W., Wang, S., Chen, Y., Zhang, X., Yang, J., Zhao, S., Ding, H. F., and Lu, D. (2010). Analysis of specialized DNA polymerases expression in human gliomas: association with prognostic significance. *Ne[illegible]o-oncolog[illegible]* **12**, 679-686. doi:10.1093/neuonc/nop074

Wu, Y., Xie, Z., Chen, J., Chen, J., Ni, W., Ma, Y., Huang, K., [illegible]ng, G[illegible] Wang, J., Ma, J., et al. (2019). Circular RNA circTADA2A promotes oste[illegible]ma pr[illegible]ession and metastasis by sponging miR-203a-3p and regulati[illegible]REB3 express[illegible]n. *Mol. Cancer.* **18**, 73. doi:10.1186/s12943-019-1007-1

Wu, Y., Yang, Y. and Xian, Y. S. (2019). HCR[illegible] inhibits ce[illegible] proliferation and invasion and promotes chemosensitivity in esophage[illegible] squamous [illegible]ell carcinoma. *Chem. Biol. Interact.* **308**, 357-363. doi:10.1[illegible]2019.05.032

Xia, W., Liu, Y., Cheng, T., Xu, T., Dong[illegible] and Hu, X. (2020). Down-regulated lncRNA SBF2-AS1 inhibits t[illegible]origen[illegible]sis and[illegible]rogression of breast cancer by sponging microRNA-143 and[illegible]ssing RRS1. *J. Exp. Clin. Cancer Res.* **39**, 18. doi:10.1186/s[illegible]046-020-1520[illegible]

Xu, S., Li, D., Li, T., Qiao, [illegible], Li, K., Guo, L. and Liu, Y. (2018). miR-494 sensitizes gast[illegible] cance[illegible] cells to TRAIL treatment through downregulation of survivin. *Cell. Phys[illegible]ochem* [illegible]1, 2212-2223. doi:10.1159/000495867

Zh[illegible]ng, J. Zhu, Y., [illegible]u, L., Yan, F. and Chen, J. (2019). miR-494 induces EndMT and [illegible]omotes the development of HCC (Hepatocellular Carcinoma) by targeting S[illegible]3/TGF-beta/SMAD signaling pathway. *Sci Rep* **9**, 7213. doi:10.1038/s41598-019-43731-4

Biology Open • Accepted manuscript

Zhang, Q., Li, Y., Zhao, M., Lin, H., Wang, W., Li, D., Cui, W., Zhou, C., Zhong, J. and Huang, C. (2019). MiR-494 acts as a tumor promoter by targeting CASP2 in non-small cell lung cancer. *Sci Rep* **9**, 3008. doi:10.1038/s41598-01[illegible]39453-2

Zhang, Y., Li, Y., Han, L., Zhang, P. and Sun, S. (2019). SBF2-AS[illegible] An oncog[illegible]nic lncRNA in small-cell lung cancer. *J. Cell. Bioc[illegible]m.* **12[illegible]**, 1542[illegible]15428. doi:10.1002/jcb.28809

Zhang, Y. T., Li, B. P., Zhang, B., Ma, P., Wu, Q. L[illegible]ing, L. and X[illegible], L. M. (2018). LncRNA SBF2-AS1 promotes hepatocellular carcinoma [illegible]tastasis by regulating EMT and predicts unfavorable prognosis[illegible] *Eur Rev Me[illegible] Pharmacol Sci* **22**, 6333-6341. doi:10.26355/eurrev_201810_16044

Zhang, Z., Yin, J., Lu, C., Wei, Y., Z[illegible]g, [illegible] and You, Y. (2019). Exosomal transfer of long non-coding RNA SBF2-AS1 e[illegible]es chemoresistance to temozolomide in glioblastoma. *J. Exp. C[illegible]n. Ca[illegible]er Res.* [illegible], 166. doi:10.1186/s13046-019-1139-6

Zhou, J., Zhang, S., Chen, [illegible] H[illegible] Z., Xu, Y., and Li, Z. (2019). CircRNA-ENO1 promoted glycolysis an[illegible] tumor progres[illegible]n in lung adenocarcinoma through upregulating its host gene ENO1. *Ce[illegible] Death Dis.* **10**, 885. doi:10.1038/s41419-019-2127-7

RETRACTED

Biology Open • Accepted manuscript

Figures

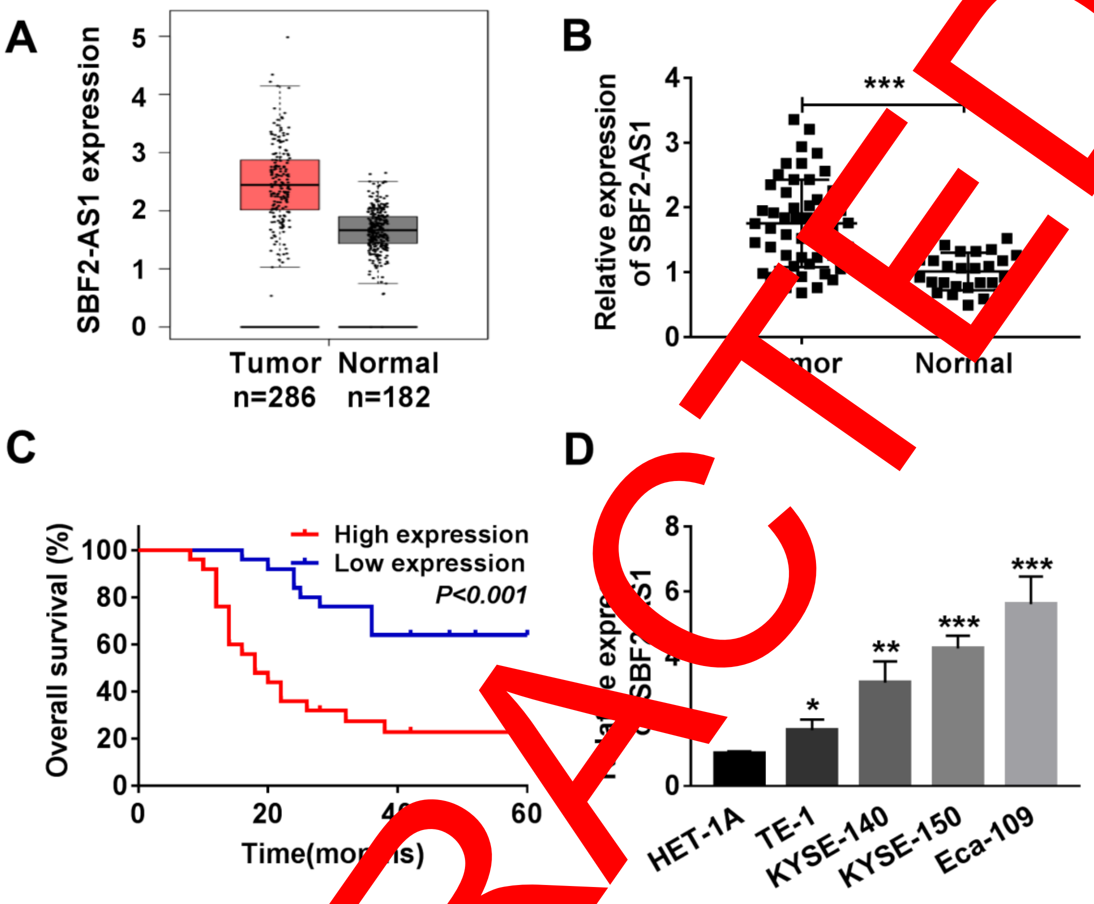

Fig. 1 SBF2-AS1 was [illegible]-regula[illegible] in ESCC. (A-B) SBF2-AS1 expression in ESCC tumors and normal tissue[illegible] was [illegible]alyzed by GEPIA2 and qRT-PCR. (C) Analysis of the overall survival rate i[illegible] ESCC patients [illegible]ith high and low level of SBF2-AS1 was conducted. (D) SBF2-AS[illegible] expres[illegible]on in E[illegible]CC cell lines (TE-1, KYSE-140, KYSE-150 and Eca-109) and huma[illegible]sophage[illegible] epi[illegible]elium immortalized cells HET-1A was measured by qRT-PCR. *[illegible]<0.05, [illegible]***P<0.001.

Biology Open • Accepted manuscript

Biology Open • Accepted manuscript

RETRACTED

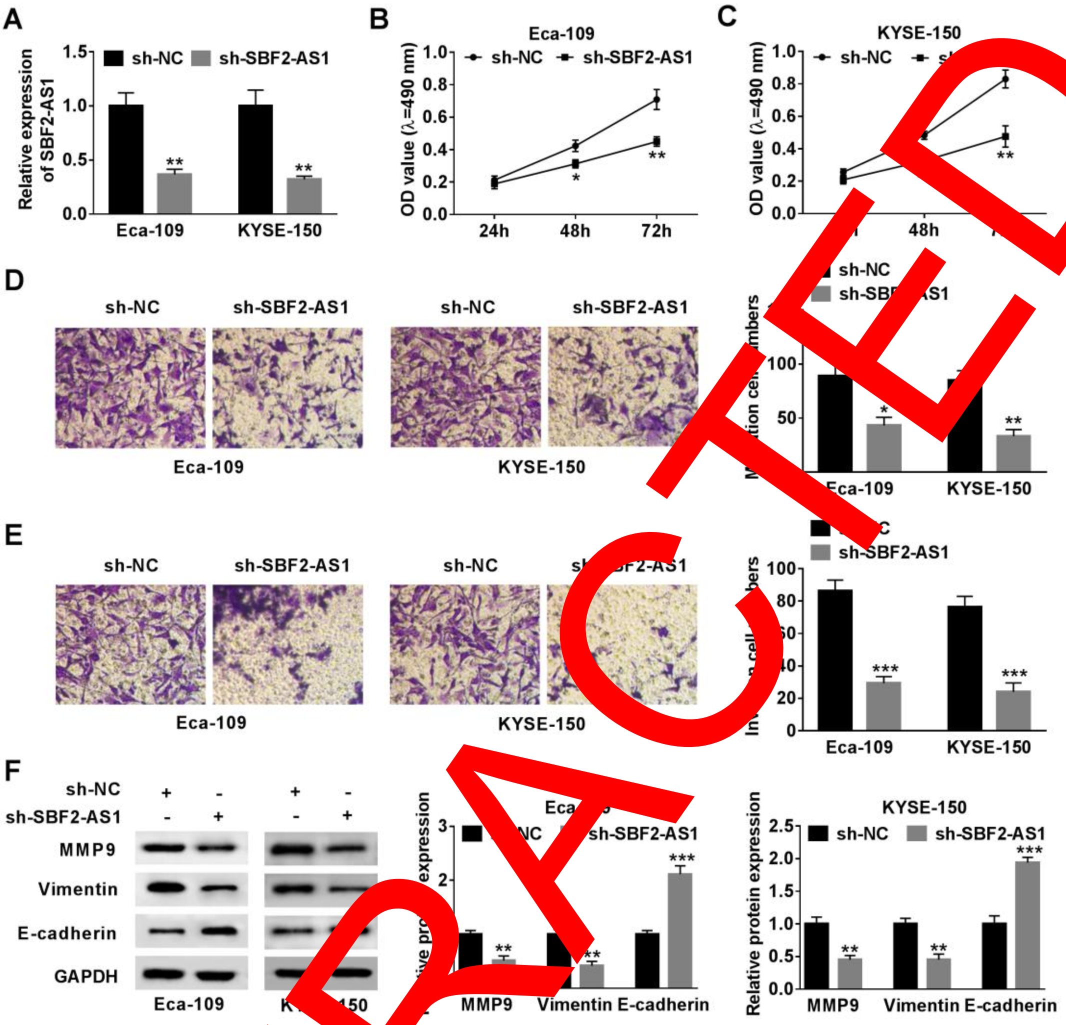

Fig. 2 SBF2-AS1 kn[illegible]ckdown inhibi[illegible]d cell proliferation, migration, invasion and EMT in ESCC cells. Eca-109 an[illegible]YSE-150 cells were stably transfected with sh-SBF2-AS1 or sh-NC. (A) [illegible]BF2-A[illegible] expression in transfected Eca-109 and KYSE-150 cells was measured by qRT-PC[illegible]) Cell [illegible]ability of transfected Eca-109 and KYSE-150 cells was measured by [illegible]TT [illegible]say. (D-E) Cell migration and invasion of transfected Eca-109 and KYSE-150 c[illegible] w[illegible]e determined by transwell assay. (F) Protein expression of MMP9, Vimentin and E-cadheri[illegible] transfected cells was assessed by western blot. GAPDH was used as internal reference. *P<0.05, **P<0.01, ***P<0.001.

RETRACTED

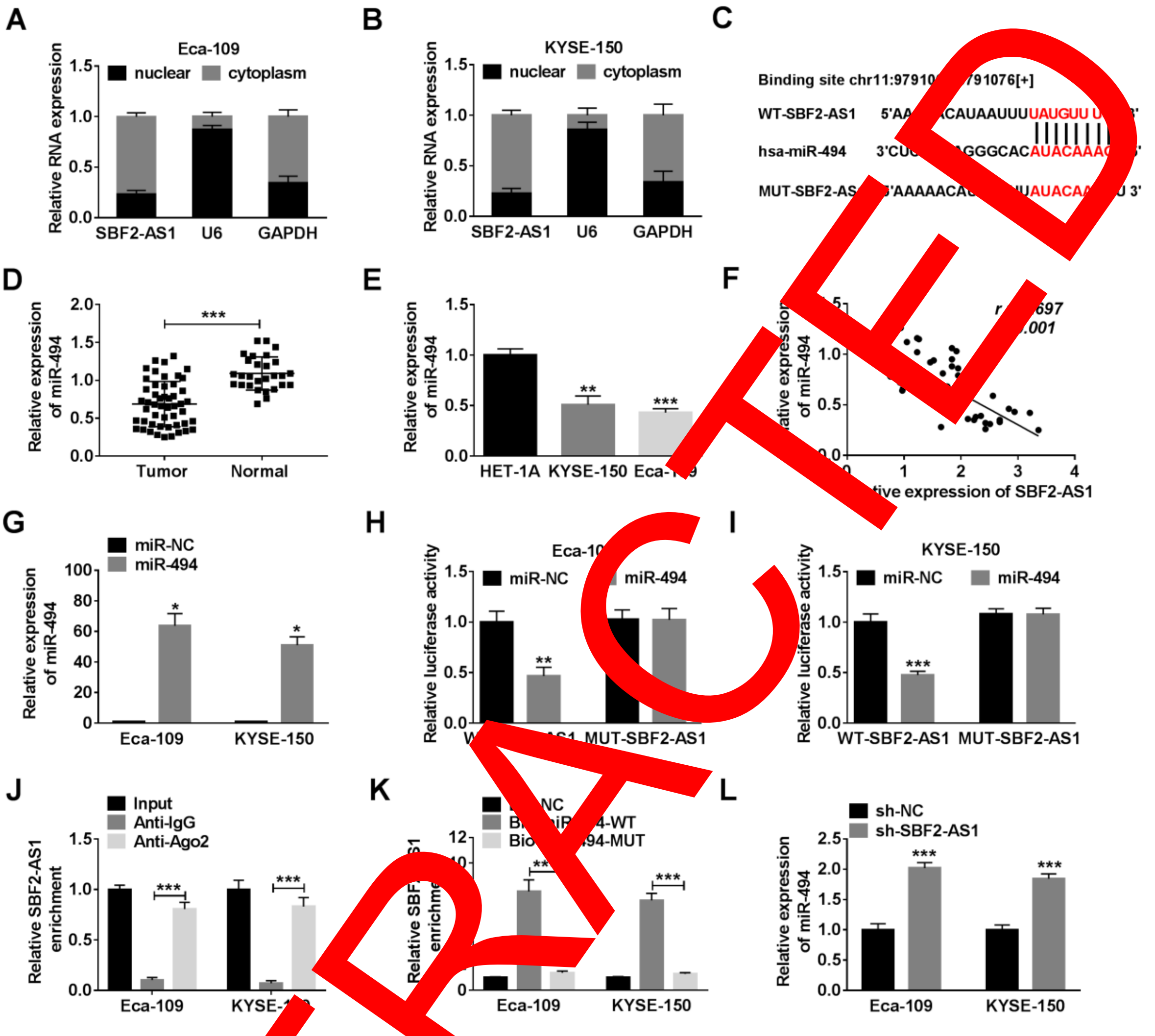

Fig. 3 SBF2-AS1 [illegible] interacted with miR-494. (A-B) The expression of SBF2-AS1, U6 and GAP[illegible]H in the nuclear [illegible]nd cytoplasm isolated from Eca-109 and KYSE-150 cells was meas[illegible]ed by [illegible]T-PCR. (C) The putative binding sites between SBF2-AS1 and miR-494 were [illegible]dicted b[illegible] Lnc[illegible]se Predicted v.2. (D) The expression of miR-494 in ESCC tumors [illegible]d norm[illegible] was measured by qRT-PCR. (E) The expression of miR-494 in Eca-109, KYS[illegible]50 and HET-1A cells was measured by qRT-PCR. (F) Analysis of the correlation between SBF2-AS1 and miR-494 was conducted (r=-0.67, *P*<0.001). (G) The expression of miR-494 in Eca-109 and KYSE-150 cells transfected with miR-494 and miR-NC. (H-I)

Biology Open • Accepted manuscript

Luciferase activity of Eca-109 and KYSE-150 cells co-transfected with WT-SBF2-AS1 or MUT-SBF2-AS1 and miR-494 or miR-NC was examined using luciferase reporter assay. (J) SBF2-AS1 enrichment in Eca-109 and KYSE-150 cells was examined by RIP assay. (K) SBF2-AS1 enrichment in Eca-109 and KYSE-150 cells transfected with Bio-NC, Bio-miR-494-WT and Bio-miR-494-MUT was detected by RNA pull-down assay. (L) The expression of miR-494 in Eca-109 and KYSE-150 cells stably transfected with sh-SBF2-AS1 and sh-NC was measured by qRT-PCR. **P*<0.05, ***P*<0.[illegible], ****P*<0.001.

RETRACTED

Biology Open • Accepted manuscript

RETRACTED

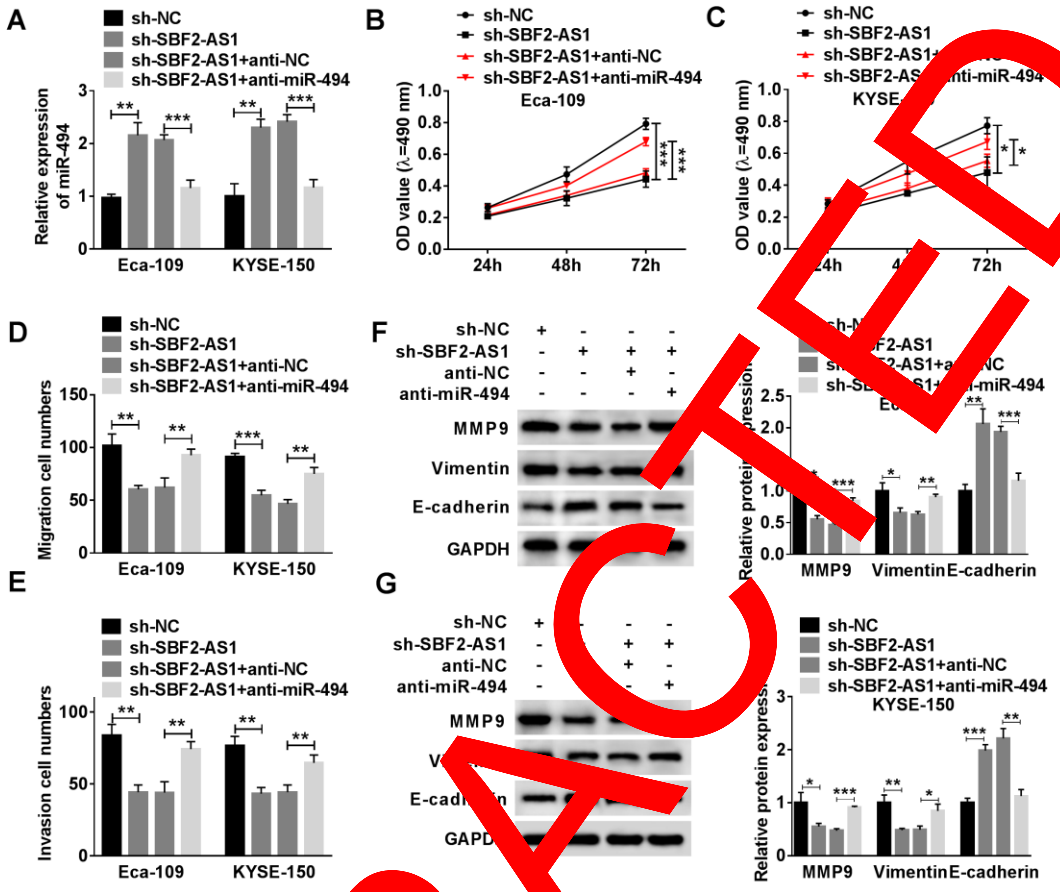

Fig. 4 MiR-494 inhibitor restores SBF2-AS1 silencing-mediated inhibition of cell proliferation, migration, invasion and EMT in ESCC. Eca-109 and KYSE-150 cells were transfected with sh-NC, sh-SBF2-AS1, sh-SBF2-AS1+anti-NC and sh-SBF2-AS1+anti-miR-494. (A) The expression of miR-494 in transfected Eca-109 and KYSE-150 cells was measured by qRT-PCR. (B-C) MTT assay was used to detect cell viability of transfected Eca-109 and KYSE-150 cells. (D-E) Cell migration and invasion of transfected Eca-109 and KYSE-150 cells were measured by transwell assay. (F-G) Western blot was applied to analyze protein expression of MMP9, Vimentin and E-cadherin in transfected Eca-109 and KYSE-150 cells. GAPDH was used as internal reference. *P<0.05, **P<0.01, ***P<0.001.

Biology Open • Accepted manuscript

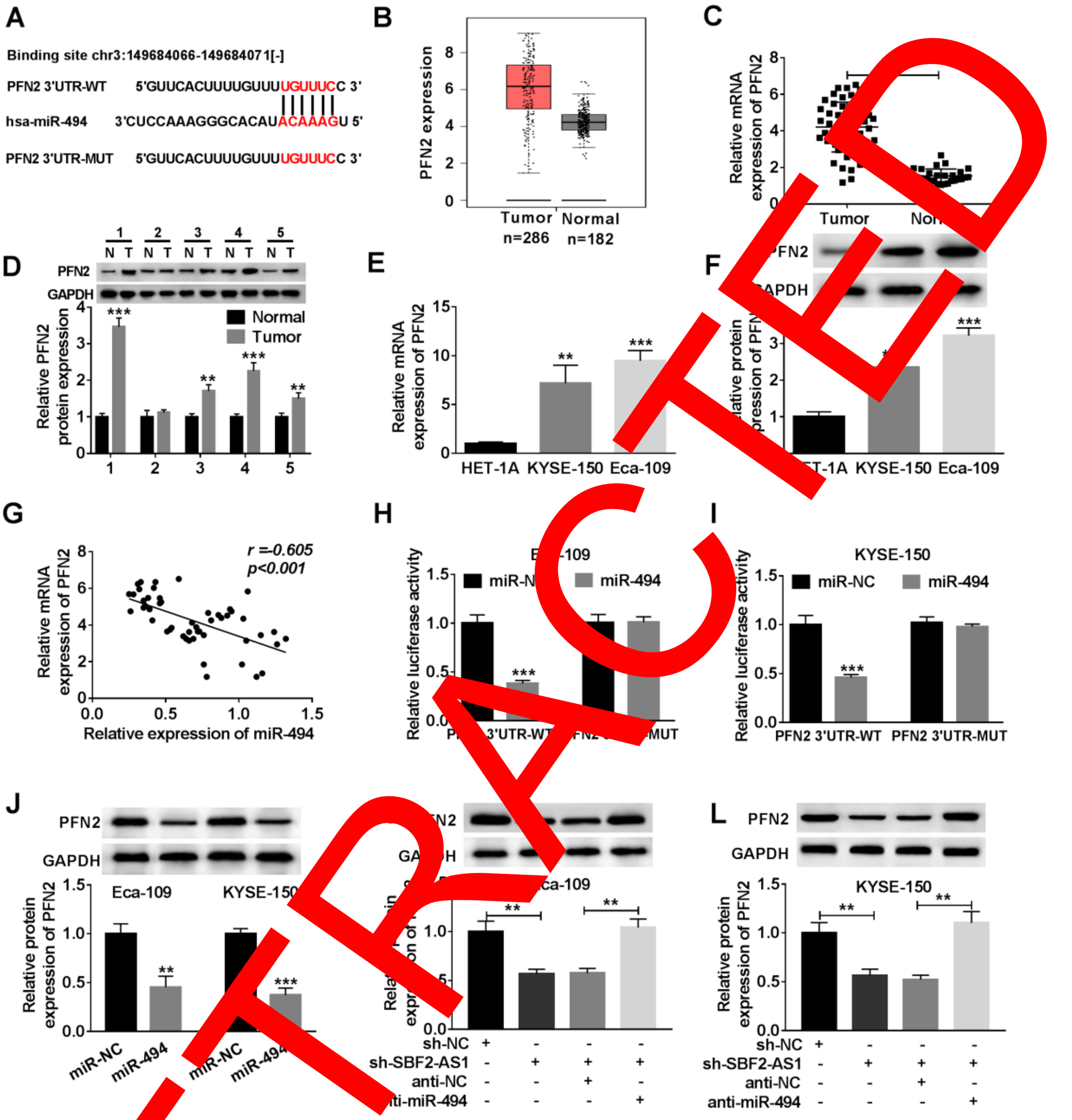

RETRACTED

Fig. 5 PF[illegible]2 was [illegible] target [illegible]f miR-494. (A) The putative binding sites between PFN2 and miR-[illegible] predict[illegible] by [illegible]arBase2.0. (B-D) PFN2 mRNA and protein expression in ESCC t[illegible]mors a[illegible] tissues were evaluated by GEPIA2, qRT-PCR and western blot, respe[illegible]ely. (E-F) The mRNA and protein expression of PFN2 in Eca-109, KYSE-150 and HET-1A cells were evaluated. (G) The correlation between miR-494 and PFN2 was analyzed (r=-0.605, *P*<0.001). (H-I) Luciferase activity of Eca-109 and KYSE-150 cells co-transfected

Biology Open • Accepted manuscript

with PFN2 3’UTR-WT or PFN2 3’UTR-MUT and miR-494 or miR-NC was evaluated by luciferase reporter assay. (J) PFN2 protein expression in Eca-109 and KYSE-150 cells transfected with miR-494 or miR-NC was assessed by western blot. (K-L) PFN2 protein expression in Eca-109 and KYSE-150 cells transfected with sh-NC, sh-SBF2-AS1, sh-SBF2-AS1+anti-NC and sh-SBF2-AS1+anti-miR-494 were analyzed. ***P*<0.01, ****P*<0.001.

RETRACTED

Biology Open • Accepted manuscript

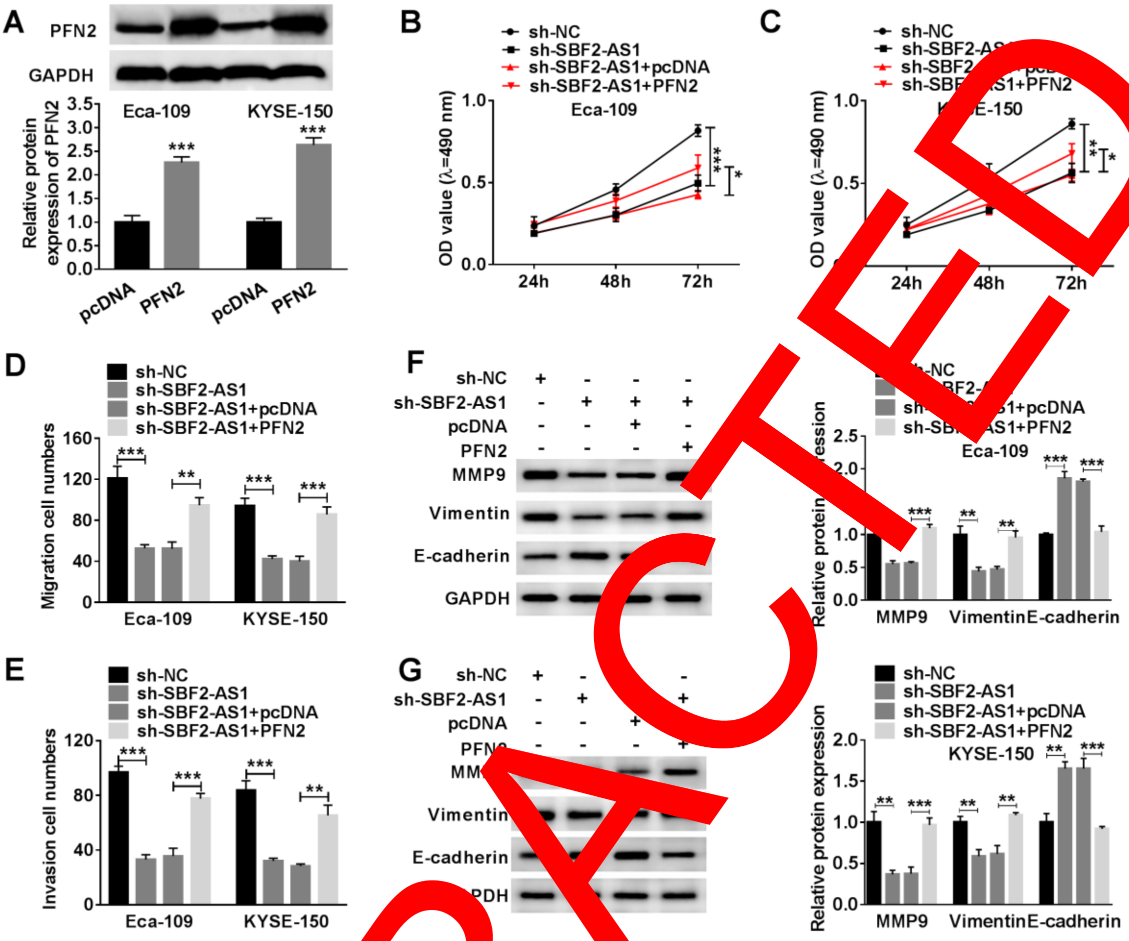

Fig. 6 PFN2 abrogated S[illegible]2-A[illegible]1 silencing-mediated inhibition on cell proliferation, migration, invasion[illegible] and EMT in [illegible]SCC. Eca-109 and KYSE-150 cells were stably transfected with sh-NC, sh-S[illegible]2-AS1, sh-SBF2-AS1+pcDNA and sh-SBF2-AS1+PFN2. (A) PFN2 prot[illegible] expre[illegible]ion in transfected Eca-109 and KYSE-150 cells was examined by qRT-PCR. ([illegible]-C) Cell via[illegible]ity of transfected Eca-109 and KYSE-150 cells was detected by MT[illegible] assa[illegible] (D-E) Ce[illegible] migration and invasion of transfected Eca-109 and KYSE-150 cells w[illegible]e [illegible]asured by transwell assay. (F-G) Protein expression of MMP9, Vimentin and E-cadheri[illegible]n transfected Eca-109 and KYSE-150 cells was evaluated by western blot. GAPDH was used as internal reference. *P<0.05, **P<0.01, ***P<0.001.

RETRACTED

Biology Open • Accepted manuscript

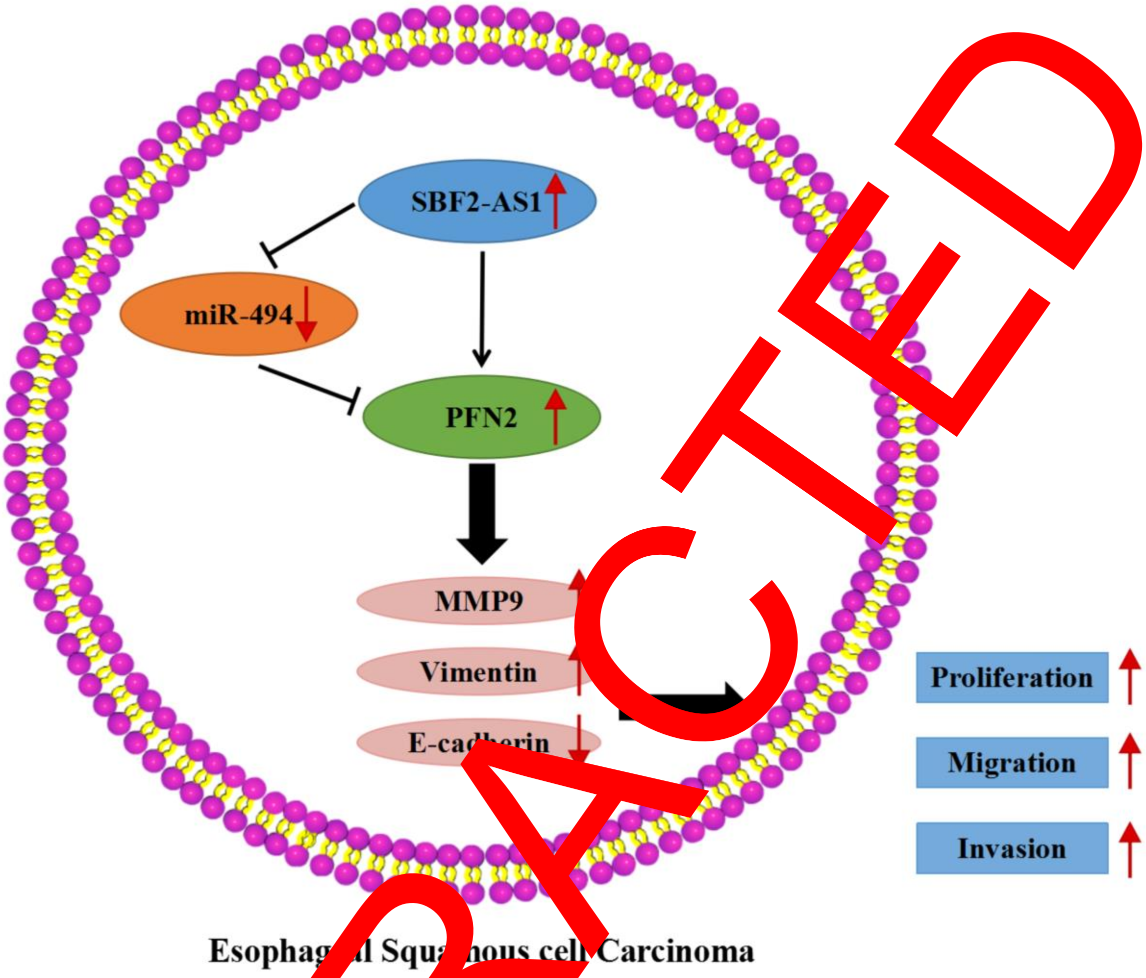

Fig. 7 The interrela[illegible]on of [illegible]F2-AS1/miR-494/PFN2 axis and cell proliferation, migration, invasi[illegible] an[illegible]MT in ESCC was illustrated.

RETRACTED

Biology Open • Accepted manuscript

RETRACTED

Supplement Table 1: Primers sequences

Name	Primer	Tm(°C)	[illegible]duct si[illegible] (bp)
SBF2-AS1	F: CACGACCCAGAAGGAGTCTAC<br>R: CCCGGTACCTTCCTGTCATA	[illegible]	[illegible]
GAPDH	F: GCACCGTCAAGGCTGAGAAC<br>R: GCCTTCTCCATGGTGGTGAA	60	226
PFN2	F: CGAGTCTCGTGGGCTACAAGGACTCG<br>R: CAACCAGGACACCCACCTCAG	60	134
U6	F: GCTTCGGCAGCACATATA[illegible]AAAAT<br>R: CGCTTCACGAATTTGCG[illegible]GTCAT	60	60
miR-494	F: GGAGAGGTTGTCCGTGT[illegible]GT<br>R: AGGTTTCCCGTGTATGTTT[illegible]	60	70

Biology Open • Supplementary information
